# Activation of proline metabolism maintains ATP levels during cocaine-induced polyADP-ribosylation

**DOI:** 10.1007/s00726-021-03065-w

**Published:** 2021-08-21

**Authors:** Sabyasachi Dash, Chandravanu Dash, Jui Pandhare

**Affiliations:** 1 Center for AIDS Health Disparities Research, Meharry Medical College, 1005 Dr. DB Todd Jr. Blvd., Nashville, TN 37208 USA; 2 School of Graduate Studies and Research, Meharry Medical College, 1005 Dr. DB Todd Jr. Blvd., Nashville, TN 37208 USA; 3 Department of Microbiology, Immunology and Physiology, Meharry Medical College, 1005 Dr. DB Todd Jr. Blvd., Nashville, TN 37208 USA; 4 Department of Biochemistry, Cancer Biology, Neuroscience and Pharmacology, Meharry Medical College, 1005 Dr. DB Todd Jr. Blvd., Nashville, TN 37208 USA; 5 Weill Cornell Medicine, Cornell University, New York, NY USA

**Keywords:** Proline oxidase, Poly(ADP-ribose) polymerase-1, Cocaine, p53, ATP

## Abstract

Cocaine is a commonly abused drug worldwide. Acute as well as repeated exposure to cocaine activates persistent cellular and molecular changes in the brain reward regions. The effects of cocaine are predominantly mediated via alterations in neuronal gene expression by chromatin remodeling. Poly(ADP-ribose) polymerase-1 (PARP-1) catalyzed PARylation of chromatin has been reported as an important regulator of cocaine-mediated gene expression. PARP-1 dependent ADP-ribosylation is an energy-dependent process. In this study, we investigated the cellular energy response to cocaine-induced upregulation of PARP-1 expression. Exposure of differentiated SH-SY5Y cells to varying concentrations of cocaine resulted in the induction of PARP-1 dependent PARylation of p53 tumor suppressor. Further analysis revealed that PARylation of p53 by cocaine treatment resulted in nuclear accumulation of p53. However, induction and nuclear accumulation of p53 did not correlate with neuronal apoptosis/cell death upon cocaine exposure. Interestingly, cocaine-induced p53 PARylation resulted in the induction of proline oxidase (POX)—a p53 responsive gene involved in cellular metabolism. Given that cocaine-induced p53 PARylation is an energy-dependent process, we observed that cocaine-induced PARP-1/p53/POX axes alters cellular energy metabolism. Accordingly, using pharmacological and genetic studies of PARP-1, p53, and POX, we demonstrated the contribution of POX in maintaining cellular energy during neuronal function. Collectively, these studies highlight activation of a novel metabolic pathway in response to cocaine treatment.

## Introduction

Cellular response to genotoxic stress is known to activate apoptotic pathways leading to cell death (Fulda et al. 2010). An important hallmark of cellular apoptosis is the activation of p53 and PARP-1 following caspase-3 cleavage (Elmore 2007). In response to genotoxic stress, p53 exerts its biological activities primarily as a transcriptional activator of genes involved in cell cycle arrest, senescence, or apoptosis (Yoshida and Miki 2010). One of the well-established mechanisms of p53-induced apoptosis is activation of pro-oxidant enzymes such as proline oxidase (POX) (Phang et al. 2008). POX *a.k.a* proline dehydrogenase (POX/PRODH) is an inner mitochondrial membrane enzyme that carries out the first step of proline catabolism (Phang 1985). First, proline can donate electrons directly for generation of ROS and the cycling of Δ^1^-pyrroline-5-carboxylate (P5C) and proline can transfer reducing potential derived either from glycolysis or the pentose phosphate shunt into ROS or ATP (Hagedorn and Phang 1986; Pandhare et al. 2006). Under stress conditions, POX generated ROS can act as a redox signal and activate p53-induced POX-dependent apoptosis (Rivera and Maxwell 2005). Other than activation of p53-induced mechanisms, ROS mediated apoptosis also induces PARP-1 and its enzymatic activity (Zhang et al. 2014). Enzymatic activation of PARP-1 is marked by subsequent addition of negatively charged poly (ADP)-ribose residues on target protein substrates, a phenomenon known as PARylation (Kraus 2015). Depletion of cellular ATP and NAD + levels are the hallmarks of cells undergoing apoptosis via PARP-1 activation (Kim et al. 2005; Kraus 2015). Although PARP-1 is predominantly known to be important in DNA repair mechanisms, the role of PARP-1 in regulating functions such as cellular energetics as well as gene transcription is increasingly being recognized. Recently, PARP-1 dependent PARylation was shown to induce genes responsible for drug-seeking behavior (Scobie et al. 2014). Under conditions of repeated cocaine administration, a global induction in PARylation was observed. The PARylated targets included an array of genes and target proteins expressed in the brain reward regions that were specifically activated by drug-seeking behavior (Scobie et al. 2014).

Cocaine is a powerful illicit drug and confers strong locomotor and reinforcing properties by increasing extracellular dopamine (DA) levels via inhibition of DA transporter (DAT) (Rocha et al. 1998). Frequent cocaine use is well characterized by compulsive drug-seeking behavior (Zahm et al. 2010; Spivey and Euerle 1990) and is known to cause alterations in various cellular metabolic pathways. Specifically, mitochondrial function and energy metabolism are affected in the brain of cocaine abusers (Cunha-Oliveira et al. 2013). Cocaine administration is accompanied by increase in brain temperature, an indicator of metabolic activation in neurons (Kiyatkin and Mitchum 2003; Kiyatkin and Brown 2006). Studies have also demonstrated oxidative stress along with a marked increase in levels of reactive oxygen species (ROS) during chronic cocaine-induced neuronal cell death in brain reward systems (Kovacic 2005; Jang et al. 2015; Dietrich et al. 2005). Cocaine-induced ROS production has been implicated in genotoxicity both in vitro and in vivo (Alvarenga et al. 2010; de Souza et al. 2014). Although, oxidative and toxic effects of cocaine have been documented, cocaine exposure primarily predisposes neurons to a reward seeking condition dependent upon gene alterations (Scobie et al. 2014; Nestler 2005). Cocaine regulates cellular gene expression by inducing chromatin remodeling via acetylation, methylation, phosphorylation as well as PARP-1 dependent PARylation (Scobie et al. 2014; Nestler 2005).

PARP-1 activity is an energy-dependent process due to its dependence on availability of NAD + (Andrabi et al. 2014). Although PARP-1 is recognized as a mediator in cocaine-induced effects, the molecular signaling triggered in response to cocaine-induced PARP-1 activation especially in the context of energetics is unclear. Therefore, in this study, we attempted to elucidate the functional relevance of cocaine-induced PARP-1 on neuronal function. By employing molecular and biochemical methods, we establish that cocaine exposure upregulates p53 and POX along with increase in ATP levels in differentiated neuronal cells. Our results also show that PARP-1 PARylates p53 in the nucleus of cocaine treated neuronal cells. Inhibition of PARP-1 dependent p53-POX axes reveals a direct link between PARP-1 activity and maintenance of POX-dependent ATP. Collectively, our results uncover a novel signaling mechanism for cocaine-induced metabolic activation involved in rewarding effects of cocaine.

## Materials and methods

### Cell culture and cocaine treatment

Human neuroblastoma cells (SH-SY5Y) were purchased from American Type Culture Collection (Manassas, VA) and were maintained in a 1:1 mixture of DMEM and Ham’s F12 medium (Gibco, USA) supplemented with 10% (v/v) heat inactivated fetal calf serum (Gibco) containing 2 mM glutamine and 1% antibiotics (penicillin–streptomycin) at 37 °C in a humidified 5% CO_2_ atmosphere. Differentiation to a neuronal phenotype was carried out as per our published method (Dash et al. 2017). Briefly, the maintenance media was replaced with serum-free medium supplemented with all-trans retinoic acid (ATRA) at a final concentration of 10 μM for 4–6 days with change of media every alternate day. For all experiments appropriate number of SH-SY5Y cells were seeded and after differentiation, were treated with cocaine concentrations from 0–100 μM. These concentrations were used since these concentrations have been suggested in literature to have physiological relevance (Van Dyke et al. 1976; Blaho et al. 2000).

### Cellular lysate preparation

*Whole cell lysate extraction* SH-SY5Y cells (1 × 10^6^) were treated with cocaine (0–100 µM) for 24 h. Cell lysates were prepared using RIPA buffer (Sigma) containing Protease Inhibitor cocktail (Sigma). Cell pellets were vortexed for 30 s and then sonicated at a setting of 10% duty cycle for 8–10 pulses (Branson Sonifier 450; Branson Ultrasonics Corp., Danbury, CT) for efficient lysis. Lysates obtained were quantified using BCA protein assay (Pierce, USA). *Nuclear and Cytoplasmic extraction* SH-SY5Y cells (1 × 10^6^) were treated with cocaine (0–100 µM) for 24 h. For lysis, with separation of nuclear and cytoplasmic components, untreated (control) and cocaine treated cells were lysed with Pierce NE-PER Nuclear and Cytoplasmic Extraction Reagents. Subcellular fractionation was performed as per the manufacturer's instructions (Pierce, USA) and samples were analyzed by western blotting.

### Western blotting

Equal amounts of cell lysates were resolved on SDS–polyacrylamide gels and transferred to nitrocellulose membranes using a semi-dry blotter (Bio-Rad). Membranes were blocked using Tris-buffered saline with 5% nonfat milk (pH 8.0; Sigma). Blots were then probed with primary p53-DO1 antibody (Sigma) and POX antibody (ProteinTech) at a dilution of 1:5000 in blocking buffer, and subsequently by a secondary antibody conjugated to horseradish peroxidase (1:10,000). All blots were washed in Tris-buffered saline with Tween 20 (pH 8.0; Sigma) and developed using the enhanced chemiluminescence (ECL) procedure (Pierce, USA). Blots were then stripped using stripping buffer (Invitrogen) at 37 °C for 15 min and probed for GAPDH as loading control using anti-GAPDH antibody (Sigma) at a dilution of 1:5000. The blots were developed using anti-rabbit or anti-mouse secondary antibodies (BioRAD, USA) at a dilution of 1:10,000. Densitometry analyses were performed using Image studio version 5.2 software (LI-COR). Data were normalized to levels of GAPDH expression.

### Immuno-precipitation

Protein-G beads (20 µl) were incubated with 1 µg of anti-PAR antibody (Trevigen) or anti-p53-DO1 antibody (SCBT) in PBS-T buffer (0.05% Tween 20 and 0.1% sodium azide) containing 1X protease inhibitor (Sigma) on a rotating mix at 4 °C for 16 h (Overnight). Next day 40 µg of freshly prepared nuclear lysate from cocaine treated cells was added to the antibody conjugated protein-G beads and incubated for 8–10 h at 4 °C. Further the samples were centrifuged at 7000 rpm for 3 min to collect the flowthrough (supernatant) and were washed three times using PBS-T (0.1% Tween 20, 0.1% sodium azide and 1X protease inhibitor) at 4 °C. Beads were then resuspended in 30 µl Laemmli buffer (Bio-Rad) with reducing agent (beta-mercaptoethanol) and were heated at 95 °C for 10 min. Samples were centrifuged at 10,000 rpm for 3 min and 30 µl of supernatant was loaded onto the gel. Samples were then electrophoresed using 4–12% gradient SDS–PAGE (Invitrogen) and transferred to nitrocellulose membranes using a semi-dry blotter (Bio-Rad). Membranes were blocked using Tris-buffered saline with 5% nonfat milk (pH 8.0; Sigma). Blots were then probed with the primary antibody p53-DO-1 (Santa Cruz) at a dilution of 1:1000 in blocking buffer. All blots were washed in Tris-buffered saline with Tween 20 (pH 8.0; Sigma) and were developed in the dark room using appropriate anti-mouse secondary-HRP conjugated antibody (Bio-Rad) at a dilution of 1:10,000. Blots were then stripped and probed for IgG light/heavy chain using respective antibodies (SCBT) at a dilution of 1:5000. Densitometry analysis was performed by ImageStudio Digits version 5.2 software (LI-COR, USA). Data obtained were normalized to IgG light/heavy chain expression relative to controls for immunoprecipitated samples.

### PARP-1 and p53 inhibition

PARP-1 activity was inhibited using ABT-888 (commercial name-Veliparib) and p53 was inhibited by alpha-pifithrin (α-PFT), a transcription activity inhibitor at a concentration of 10 µM. The concentration of 10 µM for both ABT-888 and α-PFT, was used based on several published studies that have shown this concentration to cause minimal cell toxicity (Du et al. 2016, Somnay et al. 2016, Arai et al. 2019, Balaganapathy et al. 2018). Differentiated cells were treated with either ABT-888 or α-PFT for 1 h at 37 °C. Post-treatments, cells were gently washed with 1X phosphate buffer saline (PBS) and replenished with freshly prepared differentiation media. Cocaine treatments were then performed at concentrations ranging from 0–100 µm as described above for 24 h.

### POX knockdown

POX-specific siRNAs and non-specific scrambled controls were purchased from Santa Cruz Biotechnology (Texas, USA). Differentiated SH-SY5Y cells (2 × 10^5^ cells/well) grown in 6-well culture plates were transfected with 200 nM of POX-specific siRNAs or scrambled controls using Lipofectamine 3000 (Invitrogen, USA) as per the manufacturer’s protocol. 24 h post-transfection, differentiated cells were treated with cocaine at 100 µM and further incubated for an additional 24 h at 37 °C/5% CO_2_, washed with PBS (1X) and harvested by gentle scraping for analyses. Knockdown of POX expression was confirmed by immunoblot analysis.

### Measurement of intracellular ROS

For measuring ROS levels in neuronal cells, 5 × 10^4^ SH-SY5Y cells per well were seeded in 96-well plate and were differentiated under appropriate conditions before treatment. Cells were then treated with cocaine (0–100 µM), as specified, for 24 h before analysis for ROS. 2,7-Dichlorofluorescein diacetate (DCF-DA, from Sigma) was used as an indicator of the amount of intracellular ROS. To detect intracellular ROS, treatment medium was removed, and cells were exposed to serum-free, phenol red-free medium containing 10 µM DCF-DA. Cells were exposed to the dye for 30 min in the dark to allow for equilibration. In parallel cells were solubilized with 0.5% SDS and 5 mM Tris HCl (pH 7.5). The fluorescent intensity of the lysate was determined using a spectrofluorometer (BioTek) with excitation and emission wavelengths of 485 and 530 nm, respectively. Samples were assayed in triplicate.

### Lactate dehydrogenase (LDH) assay

To determine cytotoxicity, LDH release was measured with the LDH Cytotoxicity Assay Kit (Pierce) according to the manufacturer’s protocol. 5 × 10^5^ differentiated SH-SY5Y cells were plated into 6-well plates and maintained overnight before treatment, following which the cells were treated with cocaine at various concentrations for 24 h. After treatment, the cell supernatants were collected by centrifugation. Supernatants were assayed for LDH in triplicates as per the manufacturer’s protocol. Absorbances were measured at 490 and 680 nm using a spectrophotometer (Biotek). The percentage of cytotoxicity was calculated based on the percentage difference compared with the LDH-positive control provided with the kit and fold change in LDH activity was calculated.

### Measurement of intracellular ATP levels

SH-SY5Y cells (2 × 10^5^) were plated in each well of a 24 well plate and differentiated. Cells were exposed to cocaine treatments ranging from 0–100 µM for 24 h. Post-drug treatments, the media was removed, the cells were washed with 1X PBS following which 120 µl of 1X passive lysis buffer was added to each well and incubated on a shaker incubator for 15 min at room temperature. 50 µl of lysate for each sample (drug treatment) corresponding to respective time points of each trial were plated onto a 96-well plate and processed further using ATP estimation kit (BioVision) as per manufacturer’s instruction.

### Statistical analysis

Data were analyzed using GrapPad Prism (Version 9.0.1) and expressed as mean ± SD obtained from at least three independent experiments. Significance of differences between control and treated samples was determined by one-way ANOVA for dose-dependent experiments, whereas two-way ANOVA was used for other experiments. Values of *p* < 0.05 were considered to be statistically significant.

## Results

### Cocaine exposure induces p53 expression and nuclear accumulation in differentiated neurons

**Fig. 1:**
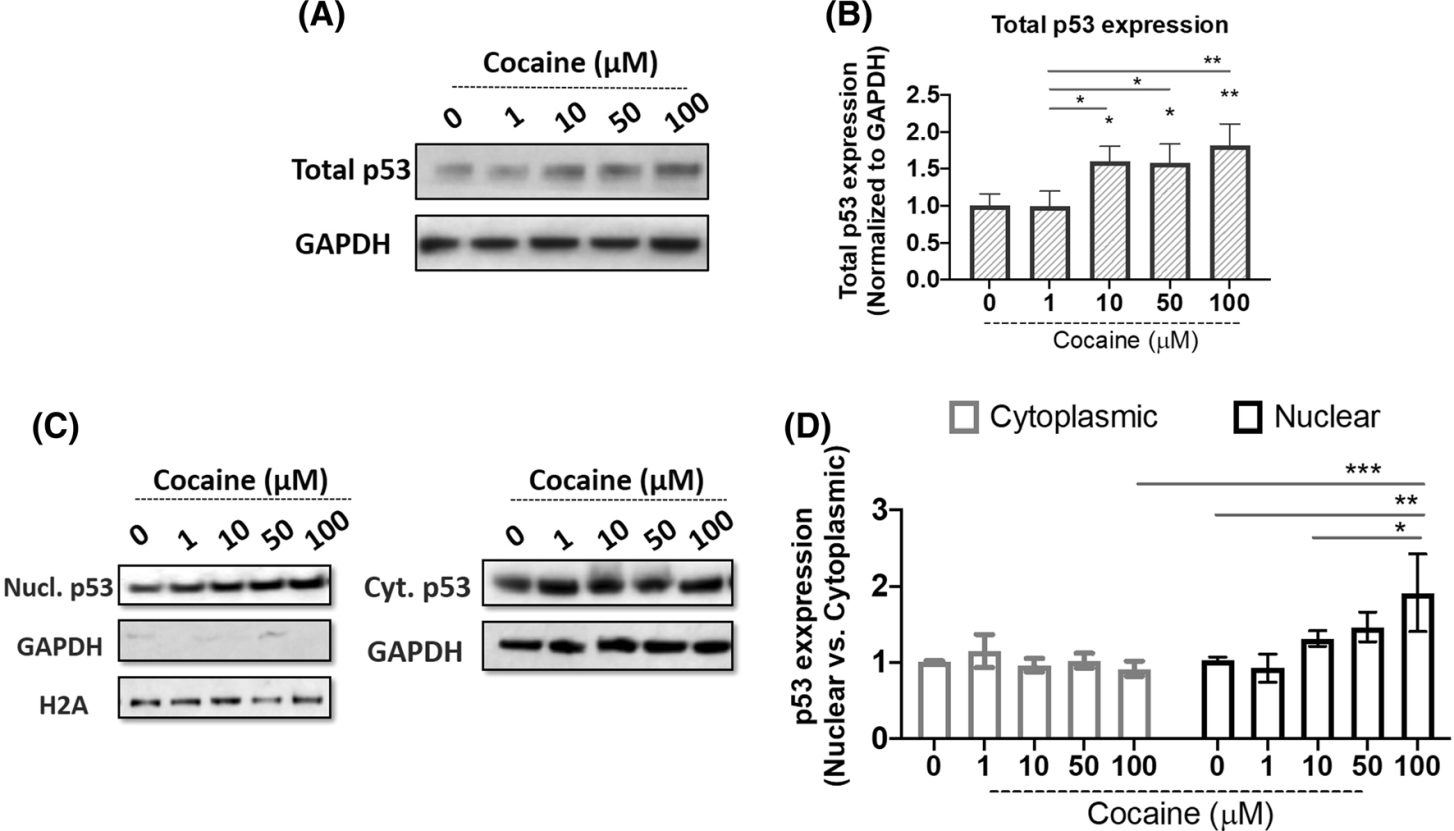
Cocaine induces nuclear accumulation of p53 in differentiated neurons. Effect of cocaine treatment on total p53 expression. Differentiated SH-SY5Y cells were treated with increasing concentrations of cocaine for 24 h. Post-treatment cells were harvested and lysed. Cell lysates were subjected to immunoblot analyses for total p53 expression. **A** Representative immunoblot of p53. **B** Densitometry analysis of p53, normalized to GAPDH. Cocaine treatment induces nuclear accumulation of p53. Post-treatment cells were harvested and processed to obtain cytoplasmic and nuclear extract as described in “Materials and methods” and further subjected to immunoblot analyses for p53 expression. GAPDH and Histone (H2A) expression were analyzed to ensure the nuclear extract purity as well as serve as a loading control. **C** Representative immunoblot of p53 in nuclear and cytoplasmic extracts. **D** Densitometry analysis of p53, normalized to H2A in nuclear extracts and GAPDH in cytoplasmic extracts. Data presented in panels **B** (one-way ANOVA, Tukey’s test) and **D** (two-way ANOVA, Tukey’s test) are mean values of three independent experiments with error bars representing SEM. *p < 0.05, **p < 0.01 and ***p < 0.001 represent statistical comparison of saline treated control (0 µM) vs cocaine treated cells

Cocaine has been shown to elicit genotoxic and cytotoxic effects leading to activation of p53 (Alvarenga et al. 2010). Studies have also shown PARP-1 regulates p53 activity in response to cellular stress (Wieler et al. 2003). Previously, we have reported the induction of PARP-1 in response to cocaine treatment without any effect on its cleavage (Dash et al. 2020, 2017). To further probe the downstream effects of PARP-1 upregulation by cocaine, we focused on the tumor suppressor p53. Differentiated SH-SY5Y cells were treated with cocaine concentrations ranging from (0–100 µM) for 24 h. Cells were then harvested and p53 levels were analyzed in cell lysates by western blotting (Fig. 1A). We observed a dose-dependent induction of p53 expression in response to cocaine treatment (Fig. 1A, B). A significant induction in p53 expression was observed at 10 µM cocaine treatment that increased further at the highest dose of 100 µM when compared to untreated controls (Fig. 1B).

In response to cellular stress, p53 accumulates in the nucleus and functions as a transcription factor mediating a multitude of cellular pathways (Inoue et al. 2005; Solozobova et al. 2009). In addition, PARP-1 is also known to stabilize p53 protein to facilitate its function (Wesierska-Gadek and Schmid 2001). Therefore, to test the functional relevance of cocaine-induced p53 expression, we analyzed the nuclear accumulation of p53. Differentiated neurons were treated with increasing concentrations of cocaine (0–100 µM). 24 h post-treatment, cells were harvested and processed for nuclear and cytoplasmic extractions. The purity of these extracts was assessed by probing for histone (H2A) in nuclear extracts and GAPDH in cytoplasmic extracts (Fig. 1C, D). We observed that p53 expression in cytoplasmic extracts of cocaine treated samples was minimally affected as a result of cocaine treatment (Fig. 1C). Interestingly, cocaine treatment resulted in a dose-dependent increase in p53 levels in the nuclear extracts with significant enhancement in the expression level of nuclear p53 with treatments of 10–100 µM cocaine (Fig. 1C, D). The expression levels of H2A in the nuclear samples (Fig. 1C) and a lack/minimal levels of GAPDH indicated the relative purity of the nuclear preparations. On the contrary, a robust expression of GAPDH was detected in the cytoplasmic extracts of cocaine treated samples. Collectively, these results demonstrate that the levels of p53 in the cytoplasm remain minimally affected; while accumulation of p53 is enhanced in the nucleus in response to cocaine treatment.

### Cocaine treatment induces p53 PARylation

**Fig. 2:**
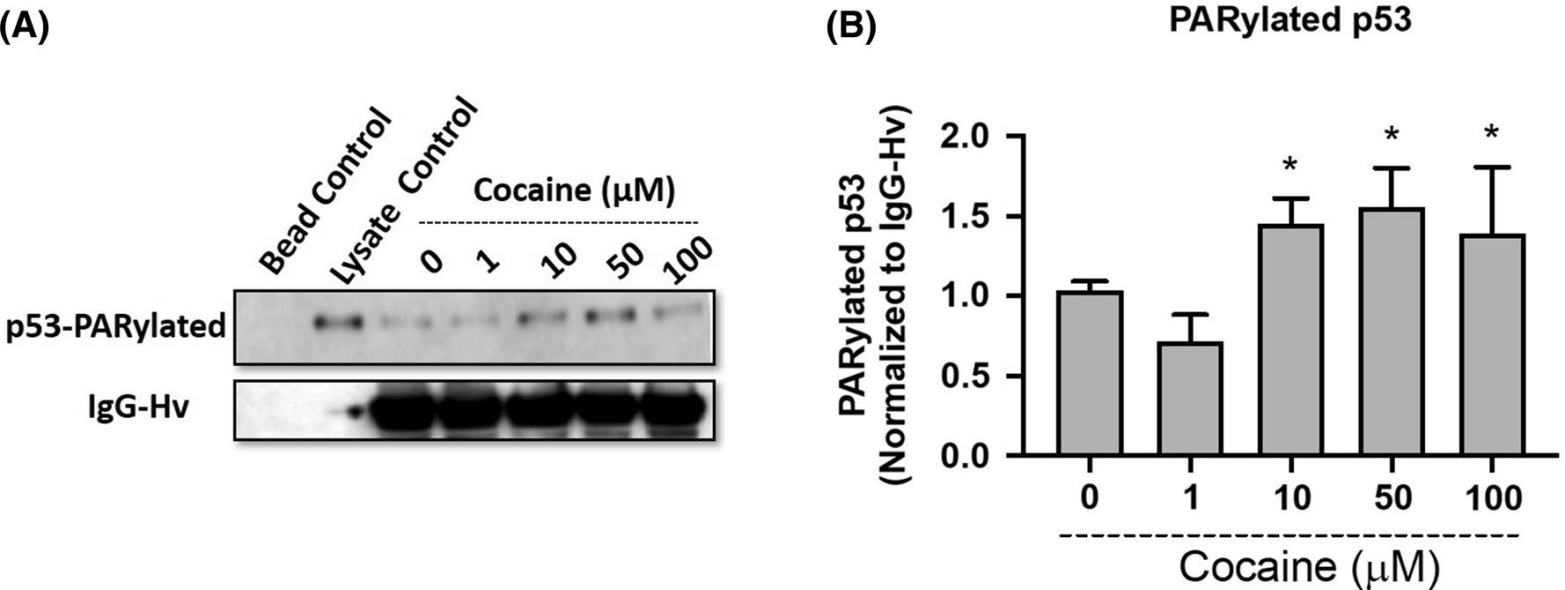
Cocaine induces PARylation of nuclear p53. Differentiated SH-SY5Y cells were treated with increasing concentrations of cocaine for 24 h. Post-treatment cells were harvested and nuclear extracts were isolated. The nuclear extracts were subjected to immunoprecipitation using an anti-PAR antibody. The proteins pulldown by anti-PAR antibody were further subjected to immunoblot analyses to detect PARylated p53 expression. **A** Representative immunoblot of PARylated p53 in nuclear extracts (*n* = 3). **B** Densitometry analysis of PARylated p53, normalized to IgG-heavy chain. One-way ANOVA, multi way analysis with Tukey’s test; * represents *p* < 0.05 for the comparison of PARylated p53 expression normalized to IgG (Hv chain) in (50–100 µM) cocaine concentrations with respect to saline treated control (0 µM)

Poly ADP-ribosylation (PARylation) is a post-translational modification that involves the addition of poly(ADP‐ribose) (PAR) to target proteins by PARP-1 (Andrabi et al. 2014). It has been reported that cellular stress can lead to activation of p53 via PARP-1 dependent PARylation (Kanai et al. 2007). Under these conditions, PARP-1 PARylates p53 in the nucleus leading to its accumulation followed by inhibition of cytoplasmic export (Kanai et al. 2007). Given that cocaine treatment resulted in increased nuclear p53 levels, we next tested whether the nuclear accumulation of p53 was due to PARylation of p53. Differentiated cells were treated with varying concentrations of cocaine and 24 h post-treatment, nuclear lysates were subjected to immunoprecipitation using anti-PAR antibody to detect the levels of PARylated p53 (Fig. 2A). A dose-dependent induction of PARylated p53 was detected in the nuclear extracts of cocaine treated cells (Fig. 2A, B). A minimal effect on p53 PARylation was observed at the lowest dose of cocaine treatment (1 µM). Densitometry analysis performed using IgG-heavy chain as a control revealed significant levels of PARylated p53 at doses of (10–100 µM) when compared to untreated control (Fig. 2B). These results suggest that cocaine treatment is associated with the nuclear translocation of p53 with subsequent PARylation by PARP-1.

### Cocaine-induced PARylated p53 induces the mitochondrial redox enzyme proline oxidase (POX)

**Fig. 3:**
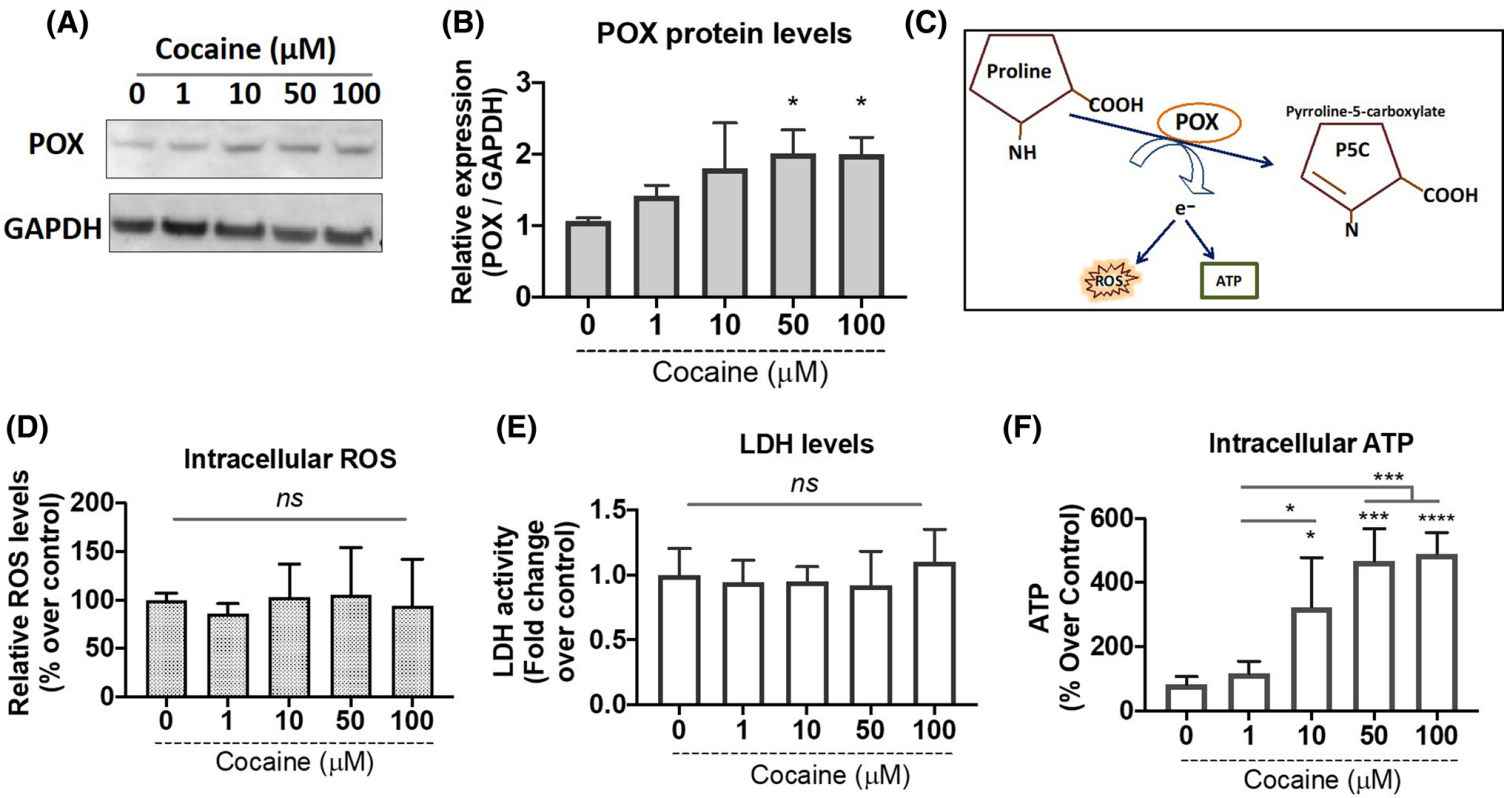
Cocaine induces POX expression concomitant to increase in intracellular ATP levels while minimally affecting intracellular ROS levels, in differentiated neurons. Differentiated SH-SY5Y cells were treated with varying concentrations of cocaine for 24 h. **A** Cells were harvested and cell lysates were subjected to immunoblot analyses for POX expression. Representative immunoblot for POX expression in response to varying concentrations of cocaine relative to saline treated control (0 µM)*.* **B** Densitometry analysis for relative intensities of POX expression normalized to GAPDH. One-way ANOVA, multi way analysis with Tukey’s test; * represents *p* < 0.05 for the comparison of relative POX expression normalized to GAPDH for 50–100 µM with respect to saline treated control (0 µM). **C** Schematic representation of POX catabolic activity generating electrons that may lead to production of either intracellular ATP or ROS. **D** Effect of cocaine concentrations on intracellular ROS. Differentiated SH-SY5Y cells were treated with varying concentrations of cocaine for 24 h. Intracellular ROS production was measured using a DCF assay as described under “Materials and methods.” Data are plotted as relative ROS levels in the saline treated control (0 µM) vs. cocaine treated cells from three separate experiments. **E** Cocaine treatment does not induce cytotoxicity. Treated cells were centrifuged and culture supernatant was collected. Cytotoxicity was measured by LDH release assay. Data represent the mean ± SEM of at least three determinations. **F** Cocaine treatments induce intracellular ATP levels. ATP levels were measured in cocaine treated differentiated neurons at 24 h. One-way ANOVA, multi-comparison analysis with Tukey’s test; *p < 0.05, ***p < 0.001, ****p < 0.0001 for the comparison of relative ATP levels normalized to total protein at respective cocaine concentrations with respect to saline treated control (0 µM)

To better understand the effects of PARylated p53 in cocaine treated cells, we focused on POX—an inner mitochondrial membrane enzyme (Phang 1985; Pandhare et al. 2006). The rationale is that PARylated p53 transactivates stress responsive genes (Kumari et al. 2014) and POX is one of the genes activated by p53 under stress conditions (Pandhare et al. 2006, 2015). To test this, we investigated whether induction and nuclear accumulation of p53 affected POX in response to cocaine treatments. We first performed cocaine treatments in differentiated neurons for 24 h. Post-treatment, cells were harvested, and POX protein expression was analyzed by western blotting. Results from this assay indicate a dose-dependent increase in POX expression in response to varying concentrations of cocaine (Fig. 3A, B). The effect of cocaine on POX levels was recorded at initial dose of 1 µM that remained induced across increasing doses when compared with untreated control (Fig. 3A). Densitometry analysis revealed a significant increase in POX expression in doses of 10–100 µM as compared to control (Fig. 3B). Collectively, these data indicate that cocaine treatment induces expression of POX in differentiated neurons concomitant to the induction of PARP-1 and p53.

POX catalyzes the first step of proline catabolism by converting proline to Δ^1^-pyrroline-5-carboxylate (P5C) (Fig. 3C). Catalysis of proline by POX generates electrons that are generally donated into the electron transport chain to generate ATP (Pandhare et al. 2009). However, under cellular stress environment, these electrons can be channeled to generate reactive oxygen species (ROS) (Pandhare et al. 2006, 2015). In addition, cocaine exposure has been shown to induce ROS mediated cytotoxicity in neurons (Dietrich et al. 2005), CD4 + T cells (Pandhare et al. 2014) as well as in cardiovascular cells (Graziani et al. 2017). Therefore, to study the effects of increased POX, we tested the effects of cocaine treatment on intracellular ROS levels in differentiated neurons. Differentiated cells were treated with varying concentrations of cocaine for 24 h. Thereafter, intracellular ROS levels were estimated by the application of peroxide-sensitive fluorescent probe 2′, 7′-dichlorofluorescein (DCF). Interestingly, cocaine treatments ranging from 0–100 µM minimally affected the levels of intracellular ROS (Fig. 3D). We also probed effects of cocaine on cellular toxicity by performing LDH cytotoxicity assay. Results from this assay reveal that cocaine minimally altered LDH release from the cells (Fig. 3E).

It is well established that ATP levels are an active indicator of cellular viability and metabolic homeostasis (Fulda et al. 2010). Additionally, activation of POX generates free electrons that can also lead to ATP production (Pandhare et al. 2009; Liu and Phang 2012). Since the differentiated neuronal cells remained viable in the presence of cocaine treatment, we speculated whether the increase in POX expression is contributing towards ATP generation rather than ROS production. Therefore, we measured the intracellular ATP levels in response to cocaine treatments. Differentiated cells were treated with a range of concentrations of cocaine for 24 h and then assayed for intracellular ATP levels. Interestingly, results from these assays reveal that cocaine treatment significantly increases total ATP levels in differentiated neurons (Fig. 3F). For instance, a minimal induction in ATP level is observed at 1 µM. However, the ATP levels were significantly enhanced with increasing cocaine concentrations ranging from (10–100 µM). Collectively, these results indicate that cocaine-induced POX expression is associated with increased levels of ATP.

### PARP-1 inhibition abrogates cocaine-induced PARylation of nuclear p53

**Fig. 4:**
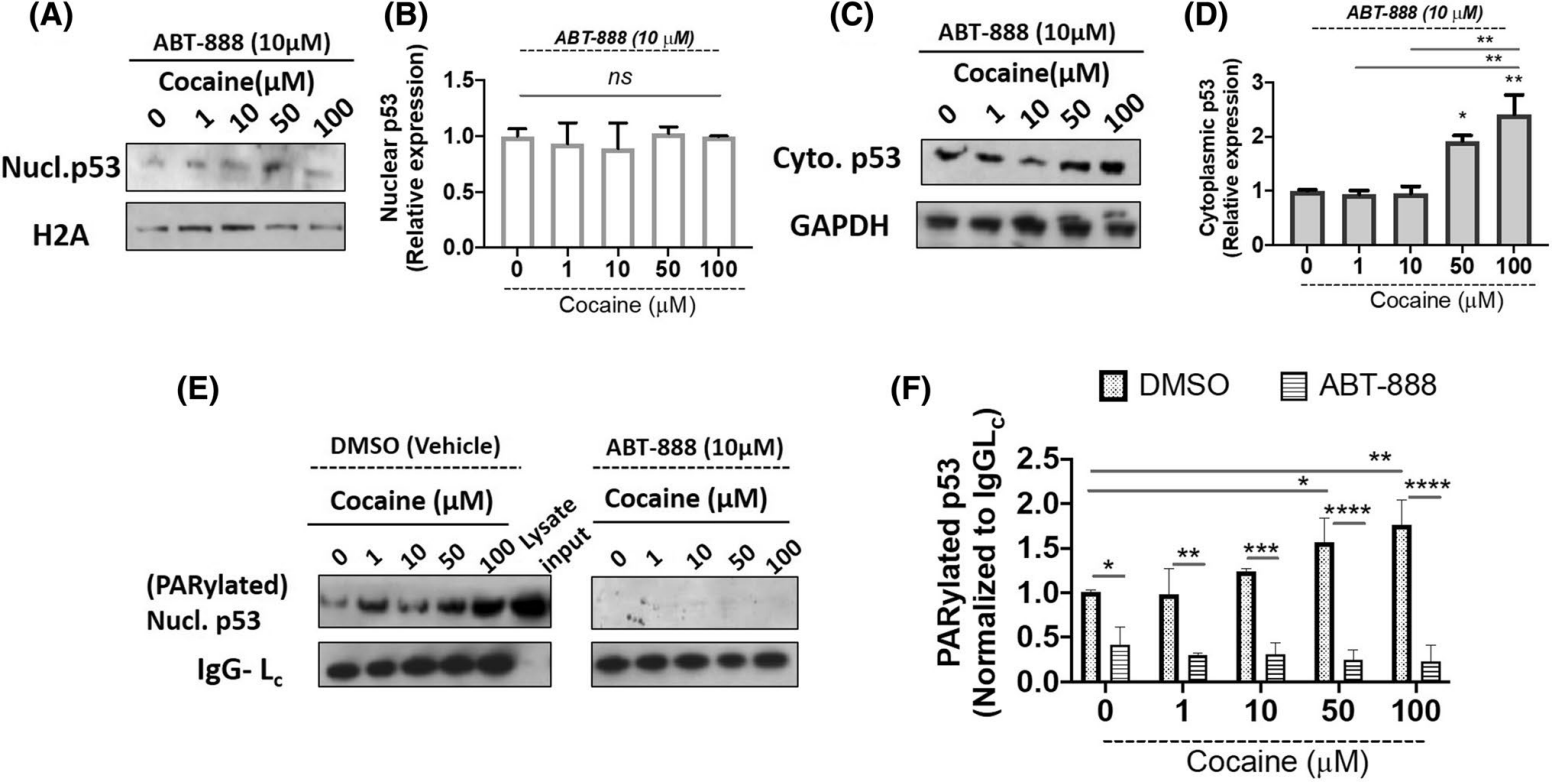
PARP-1 inhibition abrogates nuclear accumulation and p53 PARylation. Differentiated SH-SY5Y cells were pre-treated with PARP-1 inhibitor ABT-888 (10 µM) for 1 h following which the cells were treated with increasing concentrations of cocaine for 24 h. Post-treatment cells were harvested, cytoplasmic and nuclear extract were isolated and further subjected to immunoblot analyses for p53 expression. ABT-888 abrogates cocaine-induced p53 nuclear accumulation. **A**, **C** Representative blots showing the effect of ABT-888 pretreatment followed by cocaine treatment on nuclear and cytoplasmic p53 expression, respectively. Densitometry analyses of nuclear p53 (**B**) and cytoplasmic p53 (**D**) from (*n* = 3) independent experiments. One-way ANOVA, multiple comparison analysis with Tukey’s test; *p < 0.05, **p < 0.01 for the comparison of cytoplasmic p53 levels between saline (0 µM) and treated groups. ABT-888 abrogates PARylation of p53. **E** Representative western blot showing effect of cocaine on PARylated p53 in presence of DMSO (vehicle) and ABT-888 pre-treated cells with respective controls. **F** Densitometry analysis for effect of PARP-1 inhibitor (ABT-888) on levels of PARylated nuclear p53. Two-way ANOVA, multi way analysis with Tukey’s test; *p < 0.05, **p < 0.01, ***p < 0.001, ****p < 0.0001 for the comparison of relative PARylated p53 expression normalized to IgG Light chain (Lc) across varying treatments of cocaine as well as, in cocaine concentrations between the experimental groups

Our results demonstrated that PARP-1 induced PARylation of p53 enhances nuclear accumulation of p53 (Fig. 1C, D) and increased expression of POX (Fig. 3A, B). To validate the specificity of PARP-1’s effect on nuclear p53, we treated differentiated neurons with PARP-1 inhibitor ABT-888 (Veliparib) for 1 h, following which media was changed and cells were replenished with fresh differentiation media. Cocaine treatments were performed at (0–100 µM) for 24 h. Thereafter, cells were harvested and processed for nuclear and cytoplasmic extraction. Purity of extracts was analyzed using appropriate controls and p53 levels were analyzed in both cytoplasmic and nuclear extracts of cocaine treated differentiated neurons by western blot. As seen in Fig. 4A, ABT-888 pretreatment minimally changes p53 levels in the nuclear extracts (Fig. 4A). Densitometry analyses also established a minimal change in the expression levels of nuclear p53 in response to PARP-1 inhibition (Fig. 4B). Interestingly, in the cytoplasmic extracts of ABT-888 treated cells, the expression levels of cytoplasmic p53 seems to be induced at higher doses of cocaine (50–100 µM) (Fig. 4C, D). These observations are likely due to lack of PARylation and inhibition of nuclear entry due to inhibition of PARP-1 activity.

To further validate that the nuclear accumulation of p53 is dependent on PARP-1 induced PARylation of p53, we pre-treated differentiated neurons with ABT-888 followed by treatment of cocaine. The nuclear lysates were prepared and subjected to immunoprecipitation to measure PARylation of p53 (Fig. 4E). As seen in Fig. 4E, pretreatment with vehicle (DMSO) did not alter cocaine specific effects on p53 PARylation (Fig. 4E left panel). A dose-dependent increase in nuclear p53 PARylation was observed (Fig. 4F). However, inhibition of PARP-1 activity by ABT-888 significantly abrogated nuclear p53 PARylation (Fig. 4E right panel, 4F). Collectively, these data indicate that cocaine-induced PARP-1 results in PARylation of nuclear p53. Furthermore, PARylation of p53 leads to nuclear accumulation allowing its transactivation of p53-dependent genes.

### Inhibition of PARylation abrogates cocaine-induced ATP production

**Fig. 5:**
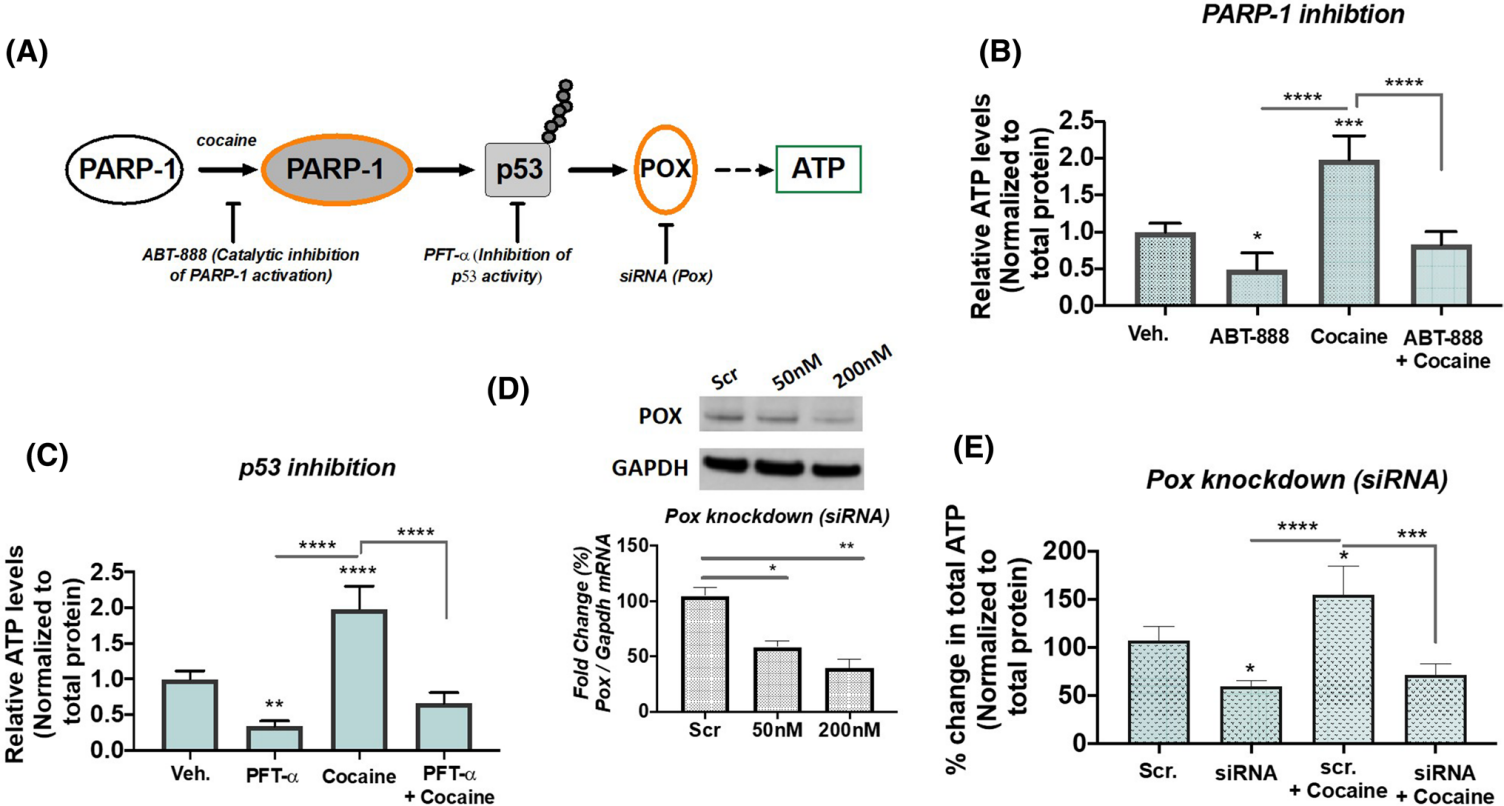
Cocaine-induced PARP-1-p53-POX signaling in differentiated neurons. **A** Schematic depicting the experimental approach to validate cocaine-induced PARP-1-p53-POX axes in maintenance of intracellular ATP levels. **B** Pharmacological inhibition of PARP-1 activity limits cocaine-induced ATP production. Differentiated SH-SY5Y cells were pre-treated with PARP-1 inhibitor ABT-888 (10 µM) for 1 h followed by treatment with cocaine at (100 µM) for 24 h and the ATP levels were measured. Data shown here are average of *n* = 3 independent trials in duplicates, one-way ANOVA, multi-comparison analysis with Tukey’s test, *p < 0.05, ***p < 0.001, ****p < 0.0001 for relative ATP levels under ABT-888 treatment. **C** Inhibition of p53 transcription activity suppresses cocaine-induced ATP production. Differentiated SH-SY5Y cells were pre-treated with p53 inhibitor α-pifithrin (10 µM) for 1 h following which the cells were treated with cocaine (100 µM) for 24 h and the ATP levels were measured. *N* = 3, independent trials, one-way ANOVA, multi-comparison analysis with Tukey’s test, **p < 0.01, ***p < 0.001, ****p < 0.0001 for the comparison of relative ATP levels under p53 transcriptional inhibition. **D** siRNA mediated POX knockdown. Differentiated SH-SY5Y cells were transfected with either POX siRNA or scrambled control siRNA as indicated for 24 h. Representative immunoblot showing the dose response inhibition in POX protein levels in differentiated SH-SY5Y cells. One-way ANOVA, multiple comparison analysis with Tukey’s test, *p < 0.05, **p < 0.001 for downregulation of normalized POX protein expression relative to scrambled control. **E** Genetic knockdown of POX prevents intracellular ATP production. ATP levels were measured in cells treated with (siRNA-POX) and without (scrambled) knockdown of POX expression for 24 h following cocaine treatment at 100 µM for 24 h. Data presented from *n* = 3 independent trials in duplicates with one-way ANOVA, multi-comparison analysis with Tukey’s test, *p < 0.05, ***p < 0.001, ****p < 0.0001 for the comparison of relative ATP levels between the experimental groups

PARP-1 cleaves the ADP-ribose moiety from NAD^+^ during poly(ADP-ribosyl)ation of specific nuclear acceptor proteins and is dependent on ATP for NAD^+^ re-synthesis (Andrabi et al. 2014). Thus, maintaining the ATP pool is essential for sustained activity in the absence of PARP-1 dependent apoptosis (Kumari et al. 2014). Our results demonstrated that cocaine induces PARP-1/p53/POX metabolic axis in the absence of cell death. Thus, we hypothesized that maintenance of cellular ATP during cocaine-induced PARP-1 is mediated via p53-dependent POX activation (Fig. 5A). To test this, we performed biochemical assays utilizing inhibitors of PARP-1 and p53 concurrent with POX knockdown studies (Fig. 5A). We used a cocaine concentration of 100 µM, since highest activation was obtained at this concentration. First, we performed ATP measurements under conditions of PARP-1 inhibition. For this, differentiated cells were treated with DMSO (control)/ABT-888 at a concentration of 10 µM for 1 h followed by cocaine treatment for 24 h. After 24 h of cocaine treatment, cells were harvested and processed for measurement of intracellular ATP levels. Data from this experiment revealed that inhibition of PARP-1 activity significantly inhibits ATP levels in the presence of cocaine, as compared to vehicle treated cells (Fig. 5B). Similarly, p53 inhibition was carried out by pretreatment with alpha-pifithrin (α-PFT) at a concentration of 10 µM for 1 h followed by cocaine treatment for 24 h. α-PFT treatment significantly reduced ATP levels when compared to control cells (Fig. 5C). Importantly, cocaine treatment failed to induce the levels of ATP in α-PFT treated cells. Finally, to establish the direct link for POX mediated ATP production in cocaine treated neurons, siRNA mediated POX knockdown was performed followed by cocaine treatment for 24 h (Fig. 5D). ATP levels measured in the cells with depleted POX were significantly lower (Fig. 5E) when compared to cells transfected with scrambled control. Further, cocaine treatment under POX knockdown conditions minimally recovered the ATP levels (Fig. 5E). Taken together these data establish a novel role of cocaine-induced PARP-1-p53-POX axis in the maintenance of cellular ATP levels in differentiated neurons.

## Discussion

Cocaine regulates cellular gene expression by inducing chromatin remodeling via acetylation, methylation, and phosphorylation of histones (Nestler 2005). Recently, PARylation of chromatin by PARP-1 has been shown as an additional chemical modification for the long term effects of cocaine (Scobie et al. 2014). Cocaine-induced alterations in cellular miRNA expression and subsequent post-transcriptional regulation of gene expression are well documented in many regions of the brain (Hollander et al. 2010; Doura and Unterwald 2016). Previously, we have reported cocaine-induced regulation of PARP-1 by miRNAs—miR-125b and miR-124 (Dash et al. 2017, 2020). Interestingly, both these microRNAs are also shown to be involved in regulation of p53 (Le et al. 2009; Liu et al. 2013; Shi et al. 2013). PARP-1 is primarily known as a DNA repair enzyme and along with p53 is essential for maintaining genomic stability (Kanai et al. 2007). Therefore, to further understand the molecular mechanisms underlying the activation of cocaine regulated PARP-1 expression, we focused on p53.

Our results show that cocaine treatment resulted in the induction of total p53 expression (Fig. 1). Several studies have suggested cocaine as a genotoxic agent (Alvarenga et al. 2010) and it is well established that p53 undergoes nuclear accumulation in response to genotoxic stress (Kanai et al. 2007; Marine 2010; Marchenko et al. 2010). Accordingly, our results revealed a dose-dependent effect of cocaine specifically on nuclear accumulation of p53. However, this increase in p53 levels was not accompanied with cytotoxicity or cell death in the differentiated cell model. Recently, it is being increasingly recognized that p53 not only controls cell cycle and apoptosis, but also regulates several pathways important in cell signaling, survival and energy metabolism (Lacroix et al. 2019; Wang et al. 2014; Humpton and Vousden 2019). In addition, p53 is known to activate cellular pathways in response to nutrient deprivation for cell survival (Humpton and Vousden 2019; Puzio-Kuter 2011). PARP-1 regulates p53 transcriptional function by inducing p53 PARylation that specifically blocks interaction of p53 with the nuclear export factor CRM1, driving nuclear localization and stimulation of p53-dependent genes (Kanai et al. 2007). PARP-1 activation is an energy-dependent process. PARP-1 uses oxidized NAD (NAD +) as a substrate to ADP ribosylate itself and various target proteins (Andrabi et al. 2014). Thus PARP-1 activity requires continuous supply of (NAD +). ADP-ribosylation is essential for various nuclear processes such as DNA repair and epigenetic regulation of gene expression (Kumari et al. 2014). Cocaine is well recognized to induce epigenetic modifications including PARylation (Scobie et al. 2014). Thus, for sustained PARP-1 activation in the presence of cocaine, maintaining the balance of energy stores is essential to prevent energy deficit and PARP-1 induced cell death (parthanatos) (Andrabi et al. 2014; Rodríguez-Vargas et al. 2019). Since p53 is a metabolic regulator and an established target of PARP-1 activity (Kanai et al. 2007; Wieler et al. 2003), we hypothesized that PARP-1 activation may result in the induction of p53 to maintain cellular energetics. Pulldown assays conducted using the nuclear lysates of cocaine treated cells confirmed PARylation of p53 (Fig. 2). To confirm the functional link between PARP-1 and p53, we probed the effects of cocaine-induced p53 PARylation in the presence of PARP-1 inhibitor (ABT-888). ABT-888, *aka* Veliparib is a well-known PARP-1 inhibitor. Inhibition of PARP-1 activity by ABT-888 attenuated cocaine’s effect on p53 PARylation and nuclear accumulation in differentiated neurons (Fig. 4). These studies suggest that cocaine induces the transcription activity of p53 via activation of PARP-1 mediated PARylation.

Next, we sought to determine the activation of downstream pathways by nuclear p53 in cocaine exposed neurons especially in the context of cellular energy. The rationale being an increase in cellular ATP levels was observed by cocaine, even in the presence of persistent PARP-1 activity (Fig. 3F). Recent studies have shown that p53 plays an important role in regulation of cellular metabolism including mitochondrial oxidative phosphorylation, glycolysis, metabolism of amino acids and lipid as well as antioxidant defense (Lacroix et al. 2019; Humpton and Vousden 2019; Puzio-Kuter 2011). p53 mediates this regulation via the induction of several target genes collectively known as p53-induced genes (PIGs). In this regard, the metabolic enzyme POX also known as p53-induced gene 6 (PIG-6) is one of the well-established targets of p53 (Rivera and Maxwell 2005; Pandhare et al. 2015). POX is an inner mitochondrial enzyme involved in proline catabolic pathway known to generate electrons (Pandhare et al. 2006). These electrons depending on the cellular microenvironment can contribute towards the generation of either ATP or ROS (Pandhare et al. 2006). Cocaine treatment had a marginal effect on levels of intracellular ROS suggesting that the electrons generated via POX may mainly channel towards ATP production. Accordingly, cocaine treatment resulted in an induction of intracellular ATP levels at 24 h (Fig. 3F). While inhibition of either PARP-1 or p53 transcriptional activity suppressed the effect on ATP. Thus, suggesting that parallel to the increase in PARP-1 induced PARylation, the pathways contributing to ATP production are upregulated to maintain cellular energy levels. In this context proline metabolism can play an important role since proline can be made easily available for ATP generation from the breakdown of extracellular matrix (ECM) (Wu et al. 2011; Phang et al. 2008, 2015). The predominant component of ECM is collagen (Kitchener and Grunden 2012). The availability of proline is alternatively regulated at the level of collagen biosynthesis, the main proline utilizing process (Albaugh et al. 2017). Interestingly, ADP ribosylation inhibits prolyl hydroxylase (Hussain et al. 1989), the key enzyme in collagen biosynthesis, thus sustained PARP-1 activation by inhibition of prolyl hydroxylase will in turn may make proline available for POX-dependent functions. Furthermore, prolyl hydroxylases are Fe + 2, ascorbate and α-ketoglutarate (α-KG) dependent enzymes belonging to the family of dioxygenases that include not only collagen prolyl hydroxylase, but also DNA and histone demethylases the enzymes that modulate epigenetic modifications (D’Aniello et al. 2019). Collagen prolyl hydroxylase has been shown to compete with DNA and histone demethylases for ascorbate and α-ketoglutarate (α-KG), affecting collagen biosynthesis and influencing metabolic epigenetics (D’Aniello et al. 2019). Moreover, cocaine is known to induce its effects primarily by epigenetic alterations (Nestler 2005), thus in this context, the inhibition of prolyl hydroxylases by PARylation could also contribute to cocaine-induced epigenetic regulation. However, whether cocaine stimulated POX expression is also regulated epigenetically by polyADP-ribosylation induced downregulation of collagen prolyl hydroxylase requires to be explored.

In addition to ECM breakdown, proline can also be synthesized from glutamine and glutamate or from arginine via its conversion to ornithine, thus proline metabolism forms a link between the TCA and urea cycles (Phang et al. 2008). Proline metabolism has been shown to contribute as an alternative energy source in conditions of nutrient deprivation (Olivares et al. 2017; Huynh et al. 2020; Pandhare et al. 2009). Moreover, the catabolic pathway of proline generates glutamate that can feed into the TCA cycle via its conversion to α-KG for ATP generation (Phang et al. 2015). Alternately, the cycling of proline to P5C and back to proline results in the generation of NAD +, which has been reported to be associated with activation of glycolysis (Liu et al., 2015). Therefore, we envisioned that NAD + generated during proline cycling may also be channeled towards catalytic activity of PARP-1 for mediating ADP ribosylation. Therefore, the activation of proline metabolism offers the cell a bioenergetic advantage during PARP-1 catalysis. Accordingly, we observed the induction of POX expression with cocaine treatment parallel to the increase in ATP levels. Silencing of POX expression decreased the ATP levels in presence of cocaine demonstrating the contribution of POX in ATP generation. Taken together, these findings indicate that PARP-1 induced PARylation of p53 activates POX for the maintenance of the energy levels in neuronal cells.

In summary, our studies uncover a mechanism essential for maintaining PARP-1 catalysis required during rewarding effects of cocaine. Furthermore, these studies highlight a novel functional role of proline metabolism in maintaining cellular energy homeostasis during PARP-1 activation.
